# Reduced Sperm Counts in Mice with *Dmd* Exon 51 Frameshift Mutations

**DOI:** 10.3390/genes17101171

**Published:** 2026-09-23

**Authors:** Leonid A. Ilchuk, Maxim A. Filatov, Daria M. Dolmatova, Iuliia P. Baikova, Ekaterina A. Varlamova, Anna V. Tvorogova, Alexandra V. Bruter, Yulia Yu. Silaeva

**Affiliations:** 1 Institute of Gene Biology, Russian Academy of Sciences, Vavilov St., 34/5, Moscow 119334, Russia; lechuk12@gmail.com (L.A.I.); baykjulia@gmail.com (I.P.B.); katerinavarlamova196@gmail.com (E.A.V.); annatvor@mail.ru (A.V.T.); aleabruter@gmail.com (A.V.B.); yulya.silaeva@gmail.com (Y.Y.S.); 2 Institute for Regenerative Medicine, Sechenov University, Moscow 119991, Russia; 3 Engelhardt Institute of Molecular Biology, Russian Academy of Sciences, Moscow 119991, Russia; sv.daria.m@gmail.com

**Keywords:** dystrophy, spermatogenesis, fertility, animal models

## Abstract

**Background/Objectives**: Duchenne muscular dystrophy (DMD) is caused by frameshift mutations in the *DMD* gene encoding dystrophin. Beyond skeletal muscle, dystrophin’s full and shortened isoforms are expressed in non-muscle tissues, including the male reproductive system, where it is reported to engage in spermatogenesis, sperm motility and overall fertility in patients and mouse models. We have previously generated two genetically modified mouse strains carrying insertions (insT or insG) in exon 51 of the *Dmd* gene to model DMD. Here, we characterize male fertility and related reproductive phenotypes in both strains. **Methods**: Fertility was assessed by mating, recording copulatory plugs, litter production and pregnancy rates over an extended breeding period. Sperm concentration and motility were quantified in vas deferens samples. Testicular dystrophin expression was evaluated by qPCR and immunofluorescence. Germ cell populations were examined by flow cytometry of testicular cell suspension; H&E histology with morphometry of tubular and peritubular compartments was carried out, along with immunofluorescence for cytoskeletal markers (dystrophin, actin, vimentin). **Results**: Both strains retained immunoreactivity for an N-terminal/rod-domain dystrophin epitope in testes, with modest nonsense-mediated decay of Dp427m transcripts and unaltered Dp71 levels. In a small breeding trial, mutant males, especially insG, produced fewer offspring despite comparable litter sizes, with fewer pregnancies after a copulatory plug. Sperm concentration in the vas deferens was approximately halved in both strains, with occasional azoospermia. Flow cytometry showed a selective decrease in the mature haploid (elongating) population in insG animals. **Conclusions**: In exon-51 frameshift models, the phenotype is lower sperm concentration in the vas deferens, to roughly half of control levels, with occasional azoospermia. InsG also fails to maintain a normal elongating-to-round spermatid ratio. Whether this fully accounts for the poorer breeding performance in the cohorts studied remains open. Overall, these findings underscore the functional role of dystrophin in spermatogenesis.

## 1. Introduction

Duchenne muscular dystrophy (DMD) is a genetic disorder caused by frameshift mutations in the *DMD* gene coding for a structural cytoskeletal protein—dystrophin (DMD). The most prominent pathological changes in DMD are associated with skeletal muscle cell death, especially in the diaphragm. Patients bearing non-frameshift variants of *DMD* typically display a milder phenotype, being diagnosed with Becker muscular dystrophy. Together with the existence of other functional isoforms of dystrophin sharing a common C-terminus, like tissue-specific Dp260/140/116 or ubiquitous Dp71 [1], this indicates the significance of its C-terminal region. Dystrophin expression is not limited to muscular tissue—other tissues expressing full or short isoforms of DMD undergo primary pathological changes in the presence of mutations in corresponding loci [2,3,4]. For instance, important roles of dystrophin isoforms in certain CNS regions [5], as well as the retina [6], have been demonstrated.

Solitary studies, dedicated to investigating effects of dystrophin deficiency on reproductive system development, have been conducted lately. Moreover, a patient with a splice site donor mutation in *DMD* who developed cryptorchidism was described [7]. Remarkably, in the same study, this phenotype is recapitulated in the mouse model with a corresponding homologous mutation. Dystrophin shorter isoform Dp71 is shown to be localized in postacrosomal and flagellar midpiece regions of spermatozoa, and disruption of its expression substantially reduces sperm motility and mouse fertility, while altering sperm morphology [8]. Despite the full Dp427 isoform being primarily expressed in muscular and cerebral tissues, earlier studies employing human *DMD* transgenic mice demonstrated that it is also weakly expressed in whole murine testes [9]. Finally, the mdx/*Utrn*^+/−^ model displays significant reductions in fertility and spermatogenesis disruptions [10] compared with the wild type and the mdx strain—this includes germ cell apoptosis and reduction in their proliferation capacity, and sperm malformations.

We have previously generated two genetically modified strains carrying frameshift mutations in exon 51 of *Dmd* by CRISPR-Cas9 [11]. These lines are distinct from mdx: they retain an N-terminally intact Dp427 species in skeletal muscle [11], whereas dystrophin protein is virtually absent in mdx. Here, we characterize male fertility and testicular phenotypes in both exon 51 frameshift strains. Mutant males show reduced pregnancy success after copulatory plugs and lower vas-deferens sperm concentration, with a more pronounced late-haploid deficit in insG, while seminiferous architecture and Sertoli morphology remain largely preserved.

## 2. Materials and Methods

### 2.1. Animals

Mutant and littermate control males maintained on a C57BL/6 genetic background for at least 4 generations, as well as wild-type CD1 females, were housed under controlled room conditions (22–24 °C temperature, 42 ± 5% humidity and 14/10 h photoperiod). Mice were given ad libitum access to standard chow and water. Twenty-four male and 24 female mice were utilized in the study. The use of animals was in accordance with the laws of the Russian Federation and was approved by the Institute of Gene Biology Bioethics Committee (approved 15 June 2025, bioethical committee conclusion #61).

### 2.2. Fertility Assessment Experiment

Two- to three-month-old sexually experienced male animals of both experimental and control groups were put in individual cages, each containing three wild-type females of CD1 background that is known for its exceptional reproductive performance. At the end of the trial, these males were included in sperm and testicular analyses. All female mates in the cages were examined for copulatory plugs every morning and evening throughout 84 days of experiment and, upon plug observation, left in the cage until 18 days post coitum. When females were found to be pregnant at this stage, they were transferred to separate cages to give birth. On the day of birth, the pups were counted, their sex was determined, the mother was returned to her mate, and the pups were transferred to a foster mother prepared in advance. Since *Dmd* is X-linked, all male offspring were assumed to be wild-type, while the female offspring were heterozygous for the *Dmd* mutation.

### 2.3. Sperm Quality

To analyze the quality of sperm in DMD model mice, sperm from the vas deferens was obtained. Adult male mice (approximately 5 months old, either after fertility trial or new cohorts) were sacrificed by cervical dislocation. Vas deferens was immediately explanted using fine scissors and forceps and placed into 300 μL of M2 medium, which was prewarmed to room temperature. Remaining fat tissue and blood vessels were removed with a 20 G needle, and the contents of the vas deferens were gently purged with forceps into a fresh 50 μL drop of M2 medium. After a 5 min incubation, 50 μL of M2 sperm-containing medium was diluted two-fold and 25 μL of suspension was placed in the Makler counting chamber (Sefi Medical Instruments Ltd., Haifa, Israel). The number of progressively motile (A- and B-types of spermatozoa with movement speeds faster or slower than 25 µm/s respectively), non-progressively motile (C-type), and immotile (D-type) sperm were counted. Live and dead spermatozoa were not distinguished among D-type. Each group was examined in batches of two males in three independent repeated trials (six male measurements per group). Sperm counts were analyzed as mean relative values per batch due to high absolute inter-batch variance.

### 2.4. Germline Flow Cytometry

Synchronously with sperm quality analysis, collected testes were subject to flow cytometry (FC) preparation and analysis workflow as described in [12,13,14]. Briefly, contents of freshly collected testes were purged by tearing the tunica albuginea with forceps into trypsin (PanEco, Moscow, Russia)/DNase I (New England Biolabs, Ipswich, MA, USA)/collagenase IV (Worthington, Lakewood, NJ, USA) solution, incubated with agitation at 35 °C, and gently pipetted a few times throughout incubation with non-germ cell suspension removal until suspension became uniform, followed by trypsin inactivation, adding more DNase I and straining the suspension through a 70 µm cell strainer. Cells were counted and aliquots (approx. 2 million cells) were stained with Hoechst 33342 (6 µg per million cells) and propidium iodide (2 µg/mL), followed by FC on CytoFlex S (Beckman Coulter, Indianapolis, IN, USA). The analysis on live cells was performed based on scattering profiles and Hoechst 33342’s unique environment-dependent fluorescence profile differences in the PB450 and Violet610 channels. At least 150,000 fluorescence events were collected per sample and analyzed following established protocols [12,13,14]. Although with this protocol a portion of somatic cells is removed during enzymatic digestion, residual somatic 2n2c cells may contribute to the dividing secondary spermatocyte gate (MII). Therefore, the round spermatid-to-MII ratio is interpreted as a relative metric under identical gating in all genotypes.

### 2.5. Histology and Staining

For dystrophin and actin staining, frozen tissue sections (10 µm thickness) were fixed in 4% PFA for 90 s, washed three times with 1 × PBS for 5 min, permeabilized and blocked with 0.03% Triton-X100 + 5% neutral goat serum in 1 × PBS for 1 h at RT, stained with 1:100 anti-dystrophin antibody (PA5-103275, Rabbit polyclonal antibody, Thermo Fisher Scientific, Waltham, MA, USA) overnight, followed by 1:200 secondary antibody (Goat Anti-Rabbit IgG H&L (Alexa Fluor^®^ 594), ab150080, Abcam, Cambridge, UK) and 1:200 phalloidin CF 448 © (Biotium, Fremont, CA, USA) for 1 h at RT, and counterstained with DAPI during penultimate 1 × PBS wash. Sections were mounted in Mowiol mountant. Vimentin was stained similarly with 1:100 anti-vimentin antibody (#5471, Cell Signaling Technology, Danvers, MA, USA). Images were acquired using a Leica STELLARIS (Leica Microsystems, Wetzlar, Germany) confocal microscope.

For H&E histology, testes with epididymides were lightly punctured and fixed in freshly prepared Davidson’s fixative (37% neutral buffered formalin, 96% ethanol, glacial acetic acid and dH_2_O in 2:3:1:3 ratio *v*/*v*) for 8 h, followed by three 2 h washes in 70% ethanol, standard histological processing steps and paraffin embedding. Sections of 4 µm thickness were obtained on a rotary microtome RMD-3000 (Medtehnikapoint, Saint Petersburg, Russia) and stained with regressive hematoxylin and aqueous-alcoholic eosin (Medix, Taganrog, Russia). Images were captured using a Nikon ECLIPSE Ti microscope (Nikon Corporation, Tokyo, Japan).

### 2.6. Quantitative PCR

Total RNA was extracted from freshly obtained testes using Lumizol reagent (Lumiprobe GmbH, Hanover, Germany) following the manufacturer’s recommendations. Approximately 1 µg of total RNA was used in reverse transcription using the RevM first strand cDNA synthesis kit (BelBioLab, Moscow, Russia) with random hexamer and oligo(dT)15 primers. followed by quantitative real-time PCR (qPCR). Measurements were performed in three technical replicates in 20 µL reaction volumes, each containing approx. 30 ng RNA equivalent of cDNA on a CFX96 C1000 Touch thermocycler (Bio-Rad Laboratories, Hercules, CA, USA). Primers used for qPCR are listed in Table 1. Expression levels were recalculated per sample relative to levels of hypoxanthine phosphoribosyltransferase (*Hprt1*) reference gene. 

### 2.7. Quantifications

Fiji 1.54s software was used for all image analyses and microscopy figure preparation. Minimal Feret diameter of seminiferous tubules were batch-measured as follows: low-resolution whole-slide H&E section images were color-deconvolved, and gaussian blur filter (1.5 px radius) was applied to the hematoxylin channel to “close” tubule walls resulting in “image A”. In parallel, a minimum filter with a radius of approximately a quarter of the smallest tubule’s radius was applied, and based on the result of this operation, point maxima (tubule centers) were detected and used later. Automatic wand tool operations were applied to “image A” based on tubule centers, spline-fitted convex hulls were selected, resulting in tubule outlines, and their morphometric characteristics were measured. Longitudinal tubule sections were excluded based on eccentricity values, and outlying measurements were discarded as misdetected with subsequent visual assessment. At least 50 tubule sections were analyzed for each sample.

Similarly, for vimentin quantifications, tubules were selected on background-subtracted confocal images; their outlines were shrunk by 10 µm to surpass the outermost cell layer and the integrated vimentin signal, as well as tubules’ morphometric characteristics, were extracted. 

For dystrophin quantifications, at least 20 arbitrary basal-layer cells (Sertoli, spermatogonia) were roughly selected manually in at least five tubules for each sample, and mean dystrophin signal intensity was calculated.

### 2.8. Statistics and Data Analysis

Statistical analyses and plotting were accomplished using GraphPad Prism 10 software (GraphPad Software LLC., Boston, MA, USA). Comparisons of pregnancy successes, mating periods, total offspring counts, sperm motility, expression levels and cytological populations, were carried on subgroup samples with males as experimental units. Sperm counts were aggregated per trial as experimental unit and analyzed as mean relative concentrations of male batches, as they strongly differed with littermate batch being analyzed. Repeated plugs, pregnancies and litters from the same male were not treated as independent biological replicates. Morphometric and immunofluorescence measurements (tubule diameter, peritubular-wall thickness, vimentin signal, basal-cell dystrophin intensity) were averaged per animal before testing, with individual tubules and cells being used to determine the mean value per animal. Two-way ANOVA (insT vs. insG and mutant vs. littermate control) with Šídák-corrected pairwise tests were used when both factors were present. Where controls were pooled, a Kruskal–Wallis test with Dunn’s correction was used. Exact biological n (where applicable) is given in each figure legend. Effects were treated as significant at *p* < 0.05.

## 3. Results

### 3.1. Fertility Trial

Over 84 days of the experiment, members of both mutant strains (n = 2 per group) yielded fewer offspring (nominal *p* = 0.039, Figure 1A) than their respective condition-matched control groups (Figure 1D). At the same time, the number of pups in each litter sired did not differ significantly between groups (Figure 1B); thus, the difference in productivity reflected reduced probability of mating success (Figure 1C), i.e., when matings did not lead to pregnancies, and not smaller litters. This particular observation could be explained by altered behavior, disruptions in spermatogenesis, reduced spermatozoa motility or ejaculation hindrance.

While the total number of observed copulatory plugs converged over time due to the limited pool of females available to each male and higher rates of unsuccessful plugs in mutant groups (Figure 1E), the mutant males required a longer period from the experiment start to produce a second and third plug than wild-type members (14.5 vs. 5 days, nominal *p* = 0.029 and 35.5 vs. 9 days, *p* = 0.057, respectively, on average) (Figure 1F).

### 3.2. Sperm Quality Is Mildly Impacted in Both Strains

Given the lower breeding output of examined mutant animals, we assessed the concentration and motility of spermatozoa in their vasa deferentia.

Sperm isolated from vas deferens revealed an approximately two-fold reduction in concentration in both mutant lines (including samples from fertility-trialed males) compared with age- and condition-matched wild-type controls (*p* < 0.05) (Figure 2A). Curiously, a few mutant members of both lines displayed complete absence of spermatozoa in the vas deferens lumen, which is consistent with a reduced number of successful pregnancies, although we cannot clearly exclude whether this kind of azoospermia was intermittent due to experiment design.

Sperm motility assessment showed a mild (and possibly negligible in effects) trend for decrease in the percentage of progressively motile spermatozoa in insG mutant strain (~35% reduction, *p* = 0.10, n = 5; Figure 2B,C).

### 3.3. Flow Cytometry and Histology Show Overall Tolerable Alterations

To further investigate the spermatogenic status of the mutant strains, FC and histological examination of testicular tissues were carried out.

In FC analysis of testicular cells (Figure 3A), we did not observe substantial alterations in meiosis in either of strains except for decreased ratio of spermatogonia to diploid preleptotene spermatocytes (Figure 3B,C,E,F). Strikingly, insG samples showed a significant reduction in the mature haploid (elongating) population, while round-spermatid numbers remained comparable to wild-type in both strains (Figure 3D,G).

The RS/MII ratio was not significantly changed in either line (Figure 3D), and cells successfully progressed through meiotic stages (Figure 3B,E), arguing against any major meiotic block. The elongating/RS ratio was reduced in insG only (Figure 3G).

H&E staining of testicular sections also revealed generally preserved seminiferous tubule architecture and the presence of all stages of spermatogenesis in both lines (Figure 4A,C and Figure S1). We hypothesized that since sperm is evacuated from tubules by peritubular myoid cell (Figure 4A, yellow arrows) contraction, and dystrophin is expressed in smooth muscle [15], their function could be compromised, contributing to sperm dilution. The peritubular layer, comprising myoid cells and fibroblasts, appeared to some extent thicker in insG members (3.90 ± 0.8 μm vs. 2.59 ± 0.6 μm in wild type, mean ± SD), with a smaller shift in insT animals (Figure 4B). The observed thickening may be a result of actin scaffold malformation, or some process akin to hypertrophy in skeletal muscle.

### 3.4. The Rod-Domain Dystrophin Epitope Is Retained in Both Strains

To robustly assess if expression of *Dmd* is retained in testes of mutant mice, a qPCR analysis of whole-testis RNA was carried out. It showed a modest reduction in Dp427m transcript levels—0.59 ± 0.08 and 0.37 ± 0.03 (mean ± SEM), respectively (*p* = 0.024 against controls)—consistent with nonsense-mediated decay and our previous observations in skeletal muscles, while expression of the short isoform Dp71 remained unchanged compared with wild-type littermates—1.02 ± 0.23 and 1.13 ± 0.004 (relative mean ± SEM). Utrophin (encoded by the *Utrn* gene) is a homolog of dystrophin and is known to partially compensate for functions of the latter [10,16]. Surprisingly, no upregulation in its expression was observed (Figure 5).

At the same time, immunodetection of Dp427 confirmed the presence of N-terminal dystrophin epitopes in testicular tissue sections from both insT and insG males and showed no significant difference among the substrains (Figure 6). Signal was observed in cells of the basal layer in all cases—spermatogonia and Sertoli cells, as well as certain interstitial cells and spermatozoal acrosomes and flagella. Average relative signal levels per basal layer cell were 1.00 ± 0.17 in wild-type, and 1.10 ± 0.20 and 0.92 ± 0.17 in insT and insG samples, respectively (mean ± SD, n = 2).

### 3.5. InsT and insG Mutations Do Not Affect Sertoli Cell Morphology

To further investigate the cellular basis for spermatogenic alterations, we examined Sertoli cell morphology by anti-vimentin staining. We have observed that Sertoli cells were not reduced in counts, and the quantity of protrusions formed by their cytoplasm, measured as vimentin signal inner to the basal layer of cells relative to seminiferous tubule perimeter, was not affected by *Dmd* mutations in our model mice (*p* = 0.53, Kruskal–Wallis test): 0.85 ± 0.10 and 0.96 ± 0.10 (mean ± SEM, n = 2) fold-change in insT and insG, respectively, relative to control (Figure 7). This contrasts previous observations in mdx mice [10].

## 4. Discussion

Frameshift mutations in the *DMD* gene lead to muscle dysfunction and the development of Duchenne muscular dystrophy. In the context of emerging gene therapy for this disease and improvement of the quality of life of these patients, the question of the impact of *DMD* mutations on reproductive function undoubtedly arises [17]. With the current go-to therapeutic toolbox, patients’ fertility status remained broadly irrelevant—since the majority of patients with DMD receive corticosteroid-based therapy, this issue has been poorly studied. However, studies have discovered a link between the reproductive system and DMD. Specifically, dystrophin deficiencies are shown to reduce fertility by altering sperm cell morphology and motility directly [8], lead to germ cell apoptosis, impair Sertoli cell expansion [10], and possibly lead to cryptorchidism [7].

Here, we studied two mouse models of DMD generated previously [11]. These two models are genetically distinguished by a single nucleotide (‘T’ or ‘G’ in insT and insG models, respectively) in exon 51, with insertion of either resulting in a reading frame shift. In an 84-day trial with two males per line, both mutant strains produced fewer pregnancies per recorded plug, more so in insG, while litter sizes were not reduced. We treat the breeding result as exploratory. Possible contributors of this effect include a spermatogenic or spermiogenic deficit, altered evacuation or storage of sperm, motility defects, and systemic effects of the more severe insG myopathy (not evaluated in this study). Therefore, we examined such testicular endpoints. 

Plug counts equalized by the end of the trial, so we do not infer a large, persistent deficit in mating opportunity. Nevertheless, mutant males were slower to produce a second and third successive plugs, although this effect may be confounded by other physiological and social factors. No mounting or intromission latencies, or ejaculation frequencies were recorded for this experiment; thus we cannot claim changes in sexual behavior.

While both mutant group male members showed reduced sperm concentration in the vas deferens, as well as somewhat (non-significantly) decreased progressive motility in insG samples of those males that did not have azoospermia, they certainly did not exhibit infertility at this level. Histological examinations did not reveal significant breakdowns in seminiferous tubule or epididymis structure of either strain, apart from slightly thicker peritubular compartment in insG mice, which is consistent with, but not proving, impeded sperm evacuation. Nor were disruptions in meiotic stages registered, based on flow cytometry analysis. Nevertheless, exclusively in insG strain, we did register a small, but statistically significant decrease in spermatogonial fraction, as part of the testicular side populations, which supports more pronounced findings in mdx strain [10], as well as two-fold diminution in elongated spermatid counts relative to round spermatid population, which could further explain their reduced sperm output and yield diminution in the fertility trial, suggesting minor spermiogenesis defect. To rule out the direct impact of Sertoli cell dysfunction on sperm elongation, we performed measurement of vimentin-rich protrusions of their cytoplasm. Surprisingly, we did not observe alterations in this Sertoli cell morphology or number, which is different from the situation in the mdx mice. The presence of actin staining with characteristic patterns in tubulobulbar complexes near sperm cell heads (Figure 6) supports this observation. Apoptosis levels were not measured directly.

Overall, the observed phenotype, which we may describe as mild yet influential, largely contrasts with mdx, where spermatogenesis is visibly affected, i.e., counts of pachytene spermatocytes and round spermatids are reduced, and testes are diminished in size. One possible explanation is that the C-terminally truncated form of Dp427, completely absent in mdx mice [10,18] is present in our model and is able to maintain some functions, as supported by previous muscle data [11]. 

Translation of the mutant Dp427m open reading frame is predicted to terminate in the hinge-3 region, leaving actin-binding domains intact and removing part of spectrin repeats 20–24. While this prediction is based on the exon-51 frameshift and on muscle data [11] (Figure S2), it is not independently confirmed by testicular anti-N-terminal Western blot in this study. Although dystrophins have been linked to mature sperm morphology via the cytoskeleton and to motility via redistribution and regulation of voltage-dependent channels [8,10], functions in haploid maturation remain incompletely defined, and we cannot assign elongation versus earlier spermatogenesis roles to particular dystrophin domains. Such a claim would need complementation or domain-specific rescue, which is a subject for a different study. Dp71 remains necessary for normal sperm morphology and motility in other models [8], and whole-testis Dp71 transcript levels were unchanged here, but whole-testis qPCR cannot place isoforms on particular germ-cell stages. Non-cell-autonomous contributions from Sertoli cells, peritubular myoid cells, interstitium or systemic physiology are not excluded. The scarcity of structural and protein-interaction studies on dystrophin in germ cells further limits interpretation.

We have previously demonstrated the massive difference in myodystrophic phenotype of our DMD model strains—shortly, insT mice display a milder phenotype compared with insG mice, especially in diaphragms, where in insG extensive fibrosis and fatty infiltration take place, with the majority of fibers being in an atrophic/myotube state [11]. At the same time, this strain shows a more pronounced effect across all experiments than the insT. This leads us to hypothesize that a more active hormonal dysregulation of spermatogenesis may take an effect in insG strain—it is shown that animals with damaged diaphragms face hypoxia due to dysfunctional breathing, which in turn leads to chronic stress and, with high levels of muscular inflammation, to overproduction of corticosterone [19,20,21]. Via hypothalamic kisspeptin signaling [22,23] the latter leads to undersecretion of gonadotropin-releasing hormone and luteinizing hormone (LH) by pituitary gland [24,25]. Inadequate LH levels in the bloodstream directly diminish testosterone production by testicular Leydig cells [26]. In this manner, a more severe myodystrophy phenotype could be linked to a more pronounced reproductive one, but direct hormonal measurements may be required to substantiate this point, as we measured neither corticosterone, LH, FSH, nor testosterone. Additionally, behavioral and strength measures that would test an insG stamina contribution are part of a separate study and are not reported here.

Overall, findings in murine models have direct implications for human DMD pathology and newly developed therapies. Many patients carry mutations similar to those in our models that truncate or partially ablate dystrophin while leaving actin-binding domains intact [27,28,29], and current exon-skipping or gene delivery approaches deliberately produce internally truncated dystrophins [30,31,32] whose composition closely resembles that in the study. Consequently, subtle impairments in sperm morphology, motility or hormonal support of spermatogenesis that remain clinically irrelevant and overlooked [17,33] in milder respiratory phenotypes could in turn become of concern once patients’ expected lifespan extends and sexual life becomes considered, underscoring the need to monitor reproductive parameters and assess potential secondary hypogonadism in treated cohorts. With this in mind, it becomes crucial to design dystrophin-restoring strategies that preserve the domains for both skeletal-muscle and germ-cell functions. 

## Figures and Tables

**Figure 1:**
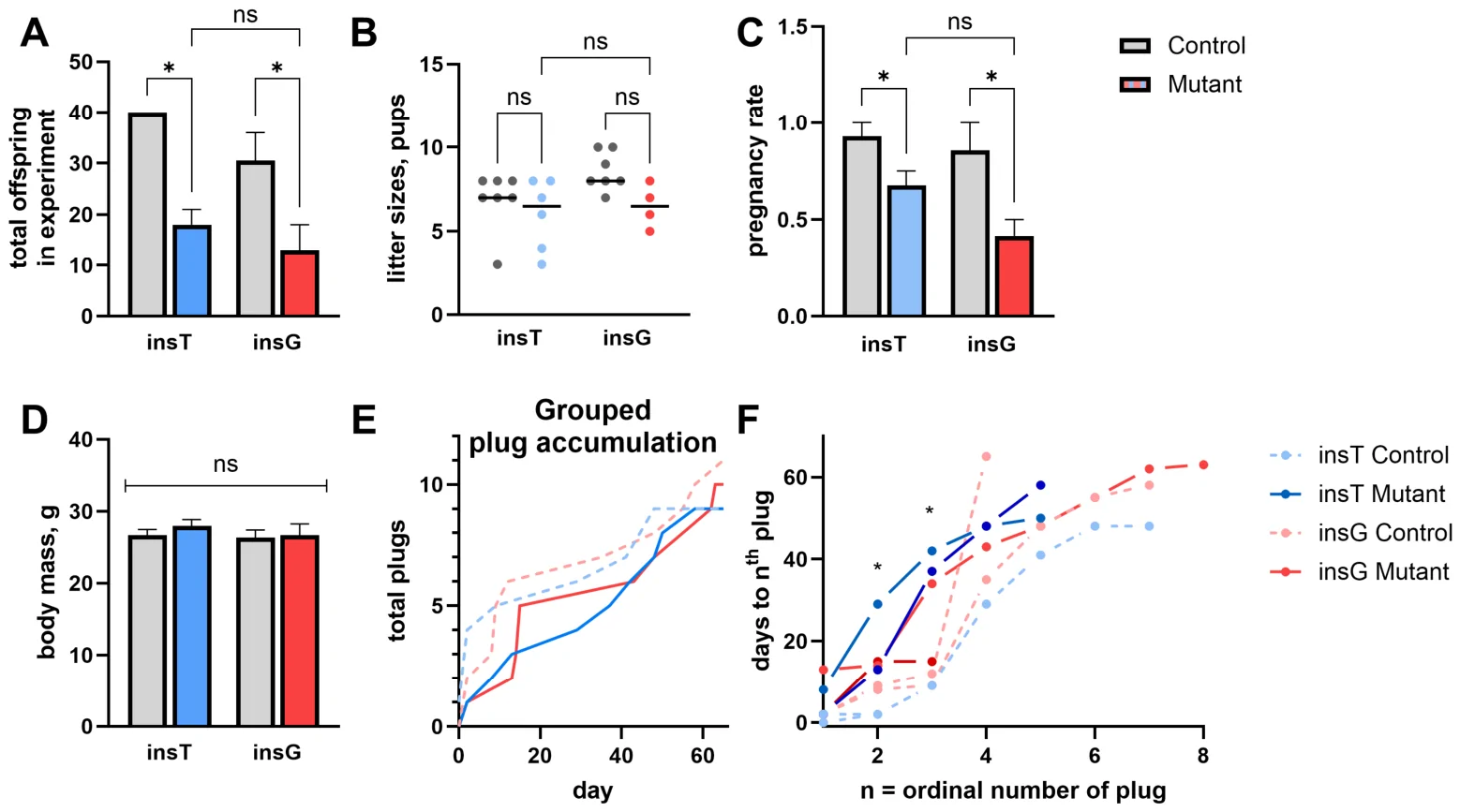
Fertility features of insT and insG males. (**A**) Outcome offspring counts in the experiment, (**B**) mean litter sizes, (**C**) successful pregnancy rate, i.e., when observed copulatory plugs resulted in pregnancy and/or birth, (**D**) body masses of experimental members, (**E**) grouped copulatory plug accumulation plot over the course of the experiment and (**F**) days it took for each consecutive plug to be produced from the start of experiment. Bars represent mean ± SEM; gray bars and dashed lines correspond to control littermate groups, while colored bars (blue for insT, red for insG) and solid lines correspond to mutant groups; n = 2 males per subgroup. * *p* < 0.05, ns *p* ≥ 0.05.

**Figure 2:**
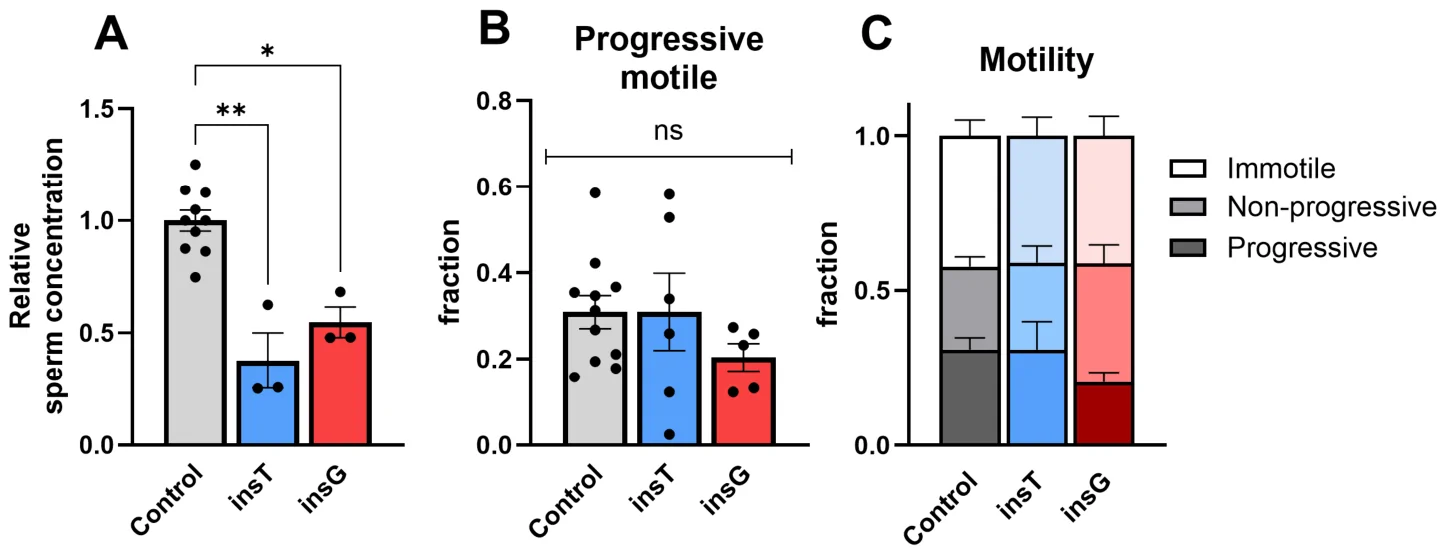
Sperm analysis in mutant (insT and insG) and control groups. (**A**) Sperm concentration change in analytes relative to control group concentrations, points are littermate trial means, 2 males per trial, (**B**) progressively motile fraction of spermatozoa in vas deferens samples, points are individual males, and (**C**) overall motility fraction composition of sperm samples with pale, intermediate and opaque bars representing immotile, non-progressive and progressive motile spermatozoa, respectively, n = 5–6, including males with assessed fertility. Shades of blue denote insT strain, while shades of red—insG. Members with complete azoospermia are excluded. Bars represent mean ± SEM. * *p* < 0.05, ** *p* < 0.01, ns *p* ≥ 0.05.

**Figure 3:**
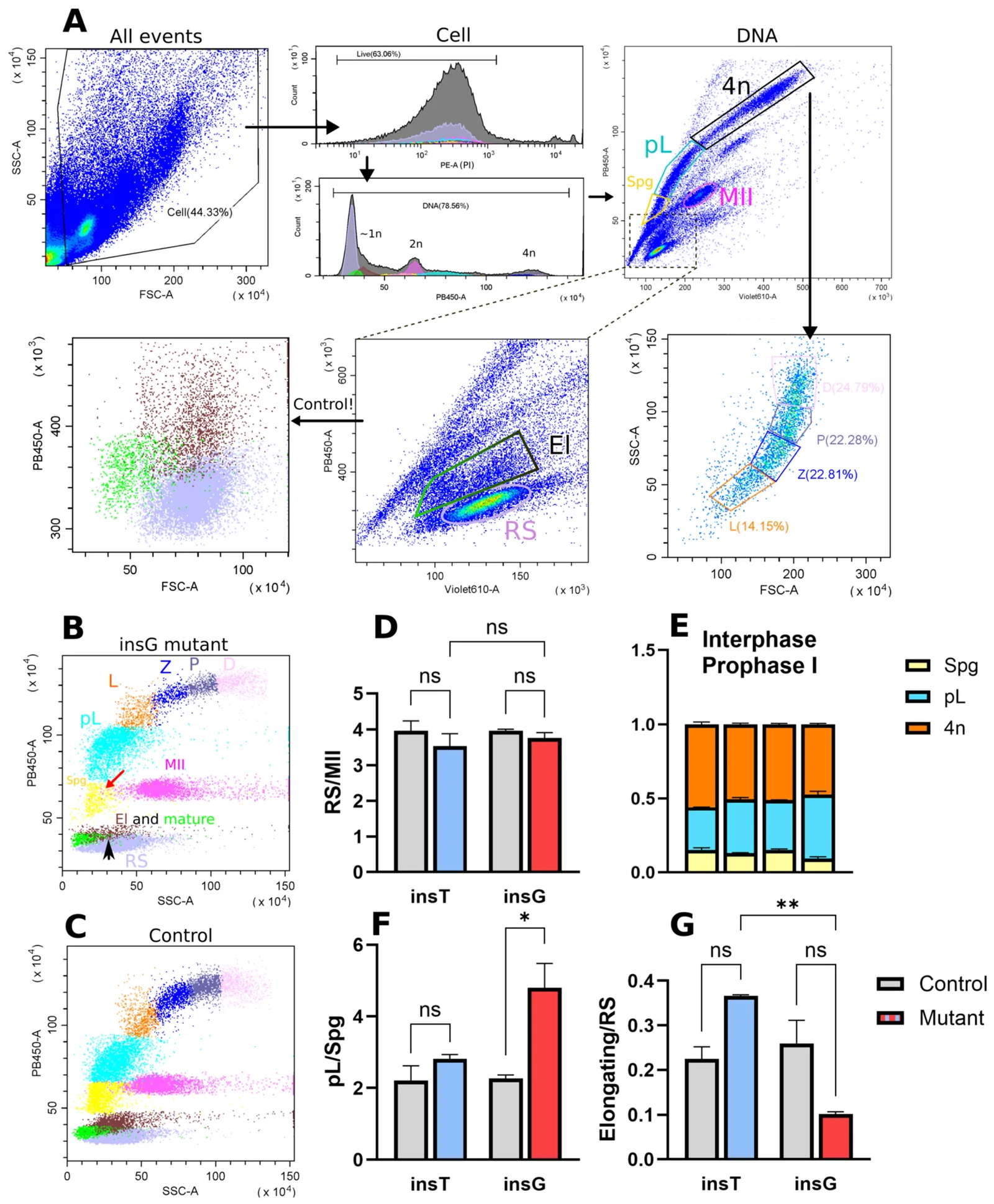
Flow cytometry (FC) workflow and population measurements. (**A**) Workflow to gate populations stained by Hoechst 33342 (Ho)—live cells are distinguished by propidium iodide (PI), and the main population is assigned to the “DNA” gate based on Ho fluorescence. Then, side populations (mainly pre-interphase spermatogonia) (Spg, yellow), preleptotene spermatocytes (pL, cyan), post-interphase primary spermatocytes (4n), secondary spermatocytes (MII, bright pink), round spermatids (RS, pale lilac) and a common set of elongated spermatids and mature spermatozoa (El, brown and green) are gated based on Ho fluorescence in 450 nm and 610 nm emission profiles. El population can further be separated, based on forward scattering (FSC), into spermatids (FSC^high^) and spermatozoa (FSC^low^). The 4n population is back-gated based on scattering profile into leptotene (L), zygotene (Z), pachytene (P) and diplotene (D) stages of prophase I, while excluding presence of pL. (**B**,**C**) summary profile of populations in sample mutant and control testis, respectively, with red arrow indicating Spg (**D**) average ratio of RS to MII, (**E**) stacked fractions of Spg, pL and 4n, (**F**) average ratio of pL/Spg and (**G**) elongating spermatozoa to RS (mean ± SEM) in control (gray bars) and mutant members (colored bars, blue for insT and red for insG) at 5 months. Total n = 4 per subgroup is displayed, including the fertility-assessed males. * *p* < 0.05, ** *p* < 0.01, ns *p* ≥ 0.05.

**Figure 4:**
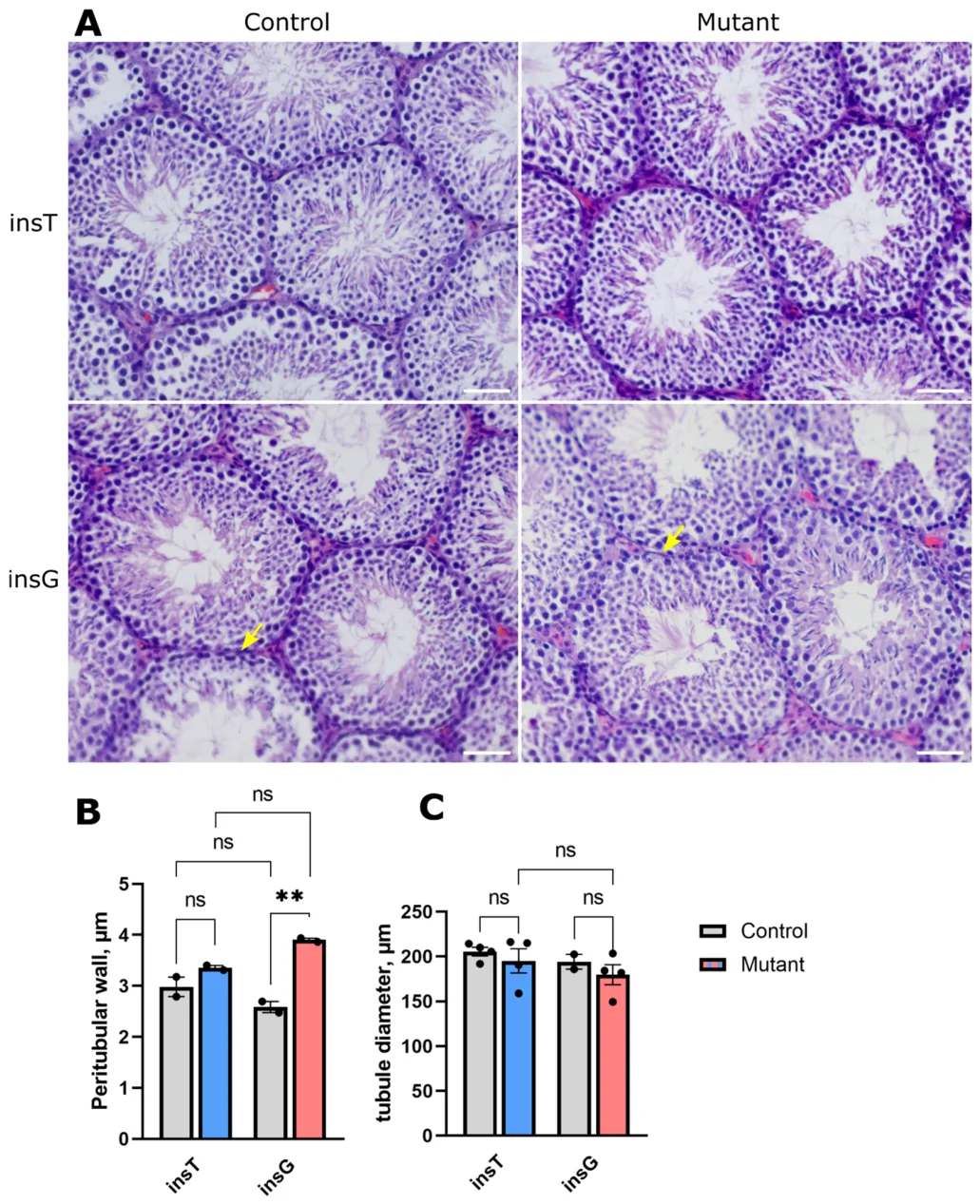
H&E staining of paraffin-embedded (PE) sections of testes and morphometric quantifications. (**A**) Samples of H&E staining of PE sections (4 µm thickness) of control and mutant (insT and insG) animals. Yellow arrows indicate nuclei of myoid cells in the peritubular wall. Scale bar: 50 µm. (**B**) Quantification of peritubular wall thickness and (**C**) tubule minimal Feret diameter in control (gray bars) and mutant (colored bars) animals, n = 2–4 males, including the fertility-assessed ones. ** *p* < 0.01, ns *p* ≥ 0.05.

**Figure 5:**
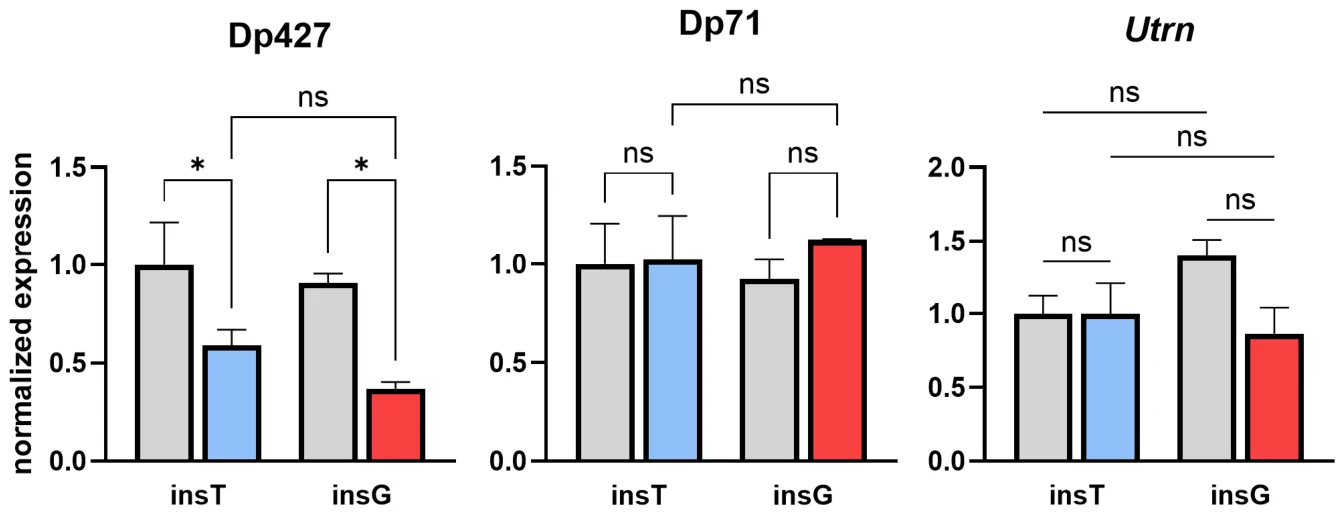
Normalized mRNA expression of dystrophin isoforms and utrophin (*Utrn*) in testes of insT and insG (blue and red bars respectively), and wild-type littermate (grey bars) mice. Quantitative RT-PCR analysis of Dp427, Dp71 and *Utrn* transcript levels in whole testes from mutant insT and insG males. Expression values were normalized to a reference gene (*Hprt1*) and are shown relative to the mean of the insT group. Bars represent group means ± SEM, n = 2 males per subgroup. * *p* < 0.05, ns *p* ≥ 0.05.

**Figure 6:**
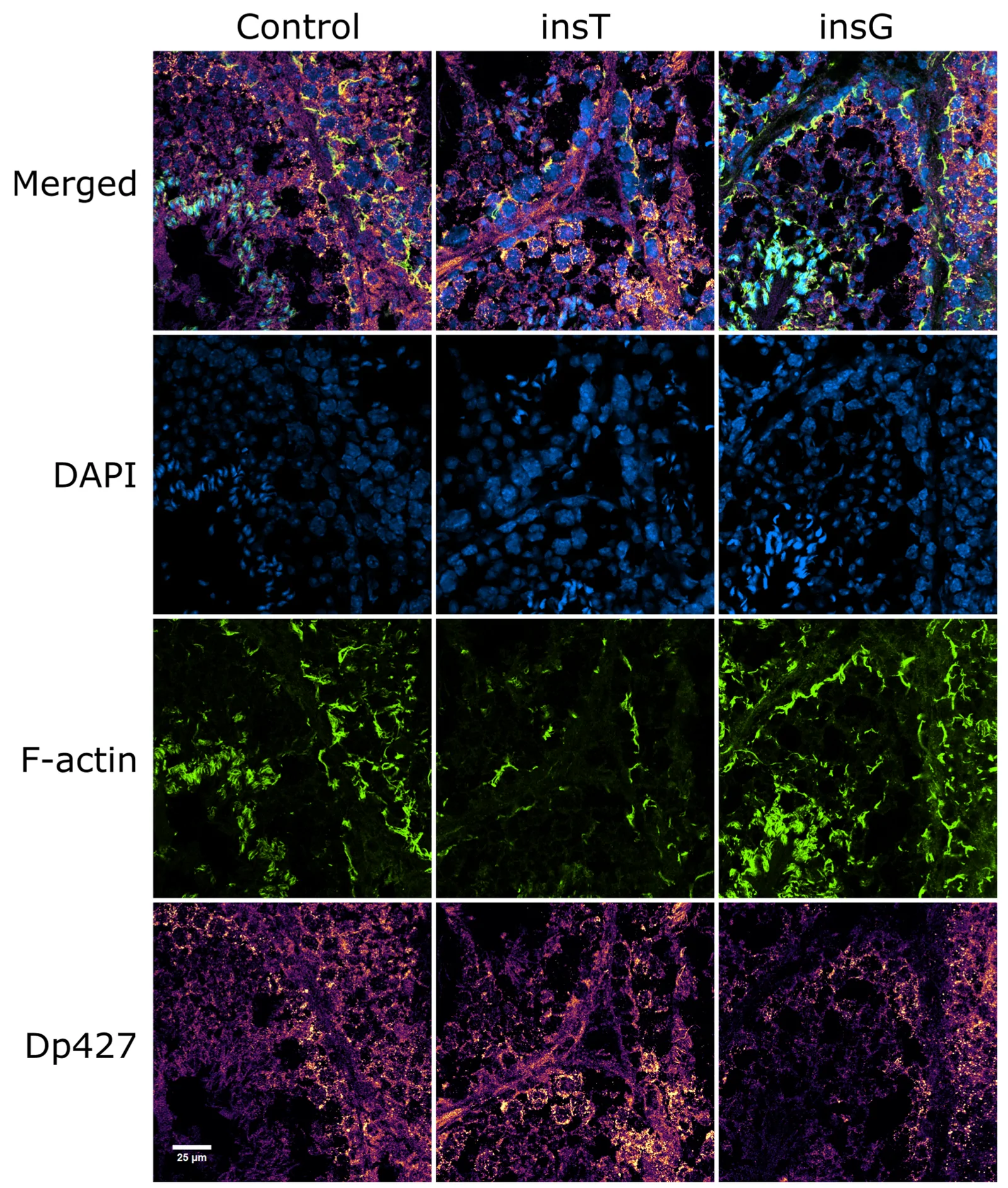
Immunostaining of frozen testicular sections of insT and insG mutant mice by DAPI (blue), phalloidin 488 conjugate (green) and anti-dystrophin antibody (purple in low-signal to bright yellow in high-signal regions), compared with control section. Scale bar 25 µm.

**Figure 7:**
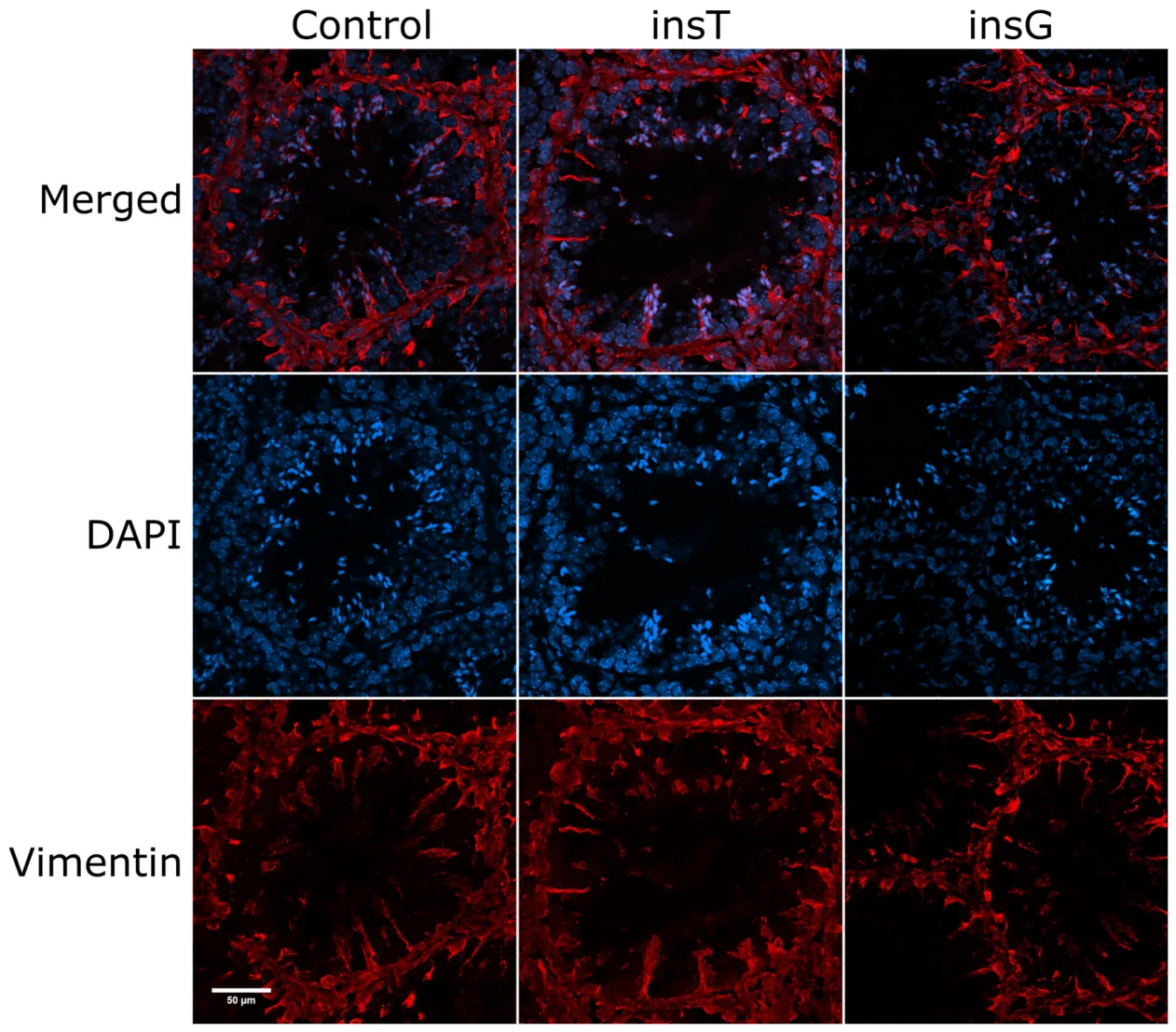
Immunostaining of Sertoli cells, distinguished by their vimentin-rich (red) cytoplasm protruding towards tubules’ deeper layers. Blue staining is DAPI. Scale bar 50 µm.

**Table 1:** List of primers used for qPCR analysis.

Target/Direction	Sequence (5′–3′)
Dp427m F	AGGAGAAAGATGCTGTTTTGCG
Dp427m R	AATTGTGCATTTATCCATTTTGTGA
Dp71 F	ACTCCTCCGCTCTAAGCGT
Dp71 R	CTTCTGGAGCCTTCTGAGC
*Utrn* F	GAACAGCAAAGGGCCACAAG
*Utrn* R	GGCCACATACTGATGAGCGT
*Hprt1* F	CAGCGTCGTGATTAGCGATGA
*Hprt1* R	GCCACAATGTGATGGCCTCC

## Data Availability

Dataset available on request from the authors.

## Supplementary Materials

The following supporting information can be downloaded at: https://www.mdpi.com/article/10.3390/genes17101171/s1, Figure S1: Seminiferous tubules’ sectors of H&E staining of paraffin-embedded sections of testes. Control (A,C) and mutant insT (B) and insG (D) tubules at stages VII–VIII. Scale bar 10 µm; Figure S2: Anti-C-terminal Western blot staining of Dp427 dystrophin of wild-type (wt) and mutant (insT and insG) muscles by ab15277 antibody (Abcam). Each lane represents individual animals.

## Abbreviations

The following abbreviations are used in this manuscript:

DMD	Duchenne muscular dystrophy
CNS	Central nervous system
FC	Flow cytometry
LH	Luteinizing hormone
