# Supplementary material for: Reduced Sperm Counts in Mice with Dmd Exon 51 Frameshift Mutations
Source: Genes (Basel). 2026 Sep 23;17(10):1171. doi: 10.3390/genes17101171 (PMC13704530; doi:10.3390/genes17101171)
Supplement: Supplementary file 1 [file genes-17-01171-s001.zip › genes-4543326-supplementary.pdf]

Supplementary figures.

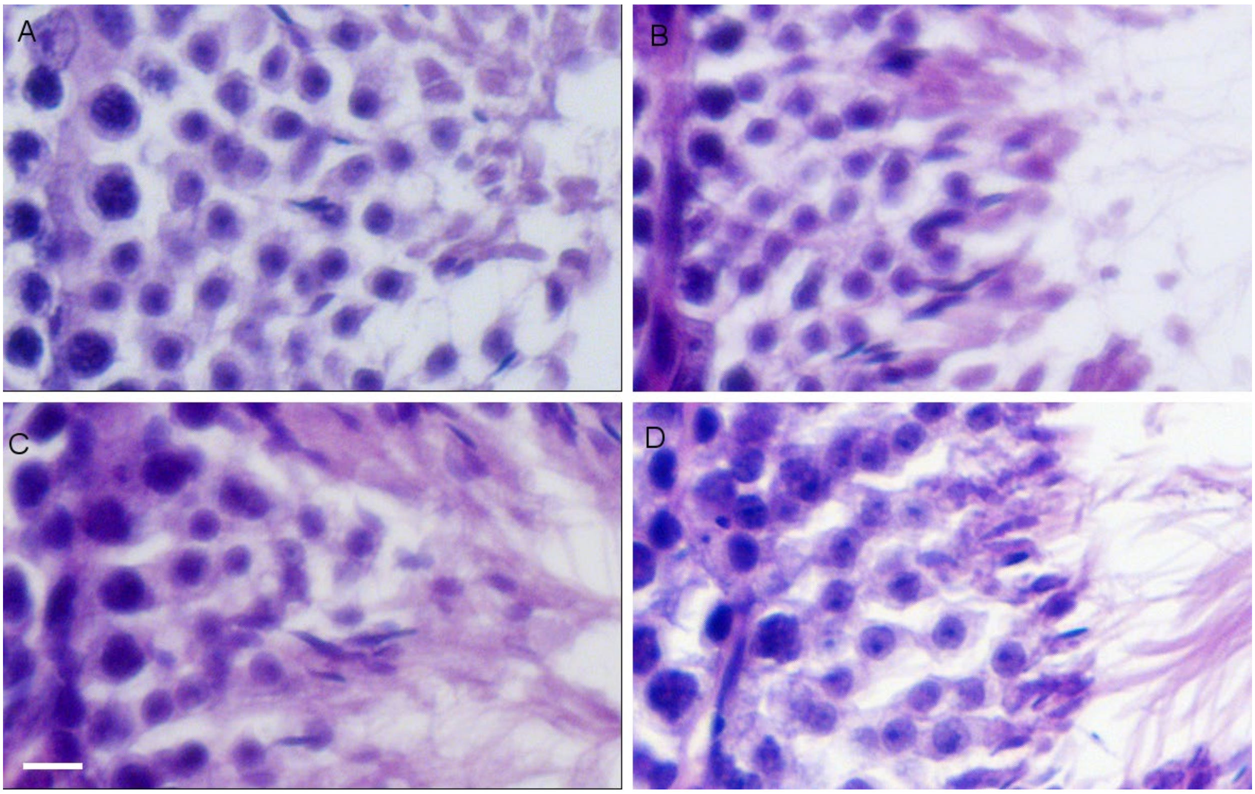

Figure S1. Seminiferous tubules' sectors of H&E staining of paraffin-embedded sections of testes. Control (A, C) and mutant insT (B) and insG (D) tubules at stages VII–VIII. Scale bar 10 µm.

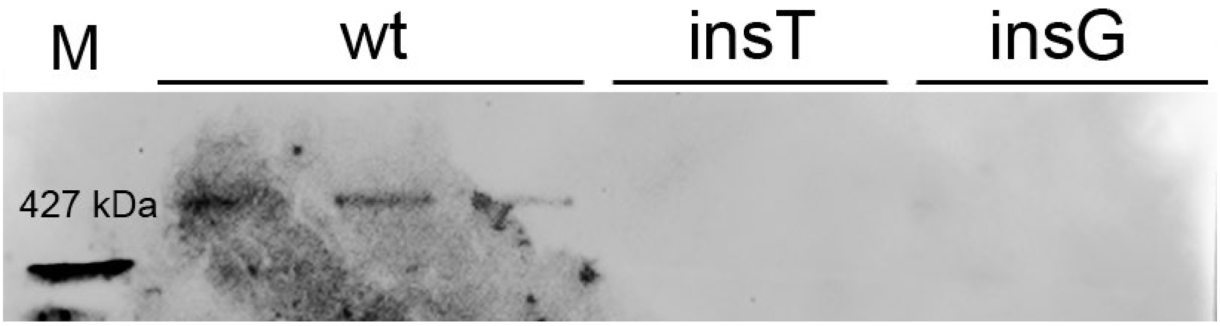

Figure S2. Anti-C-terminal Western blot staining of Dp427 dystrophin of wild-type (wt) and mutant (insT and insG) muscles by ab15277 antibody (Abcam). Each lane represents individual animals.
